# DiGeorge Syndrome Diagnosed at Age 38: Challenges in Low-resource Settings and Implications of a Missed Diagnosis

**DOI:** 10.1210/jcemcr/luae136

**Published:** 2024-07-24

**Authors:** William Kuenstner, Suthee Rapisuwon, Leila Shobab

**Affiliations:** Division of Endocrinology, Department of Medicine, MedStar Georgetown University Hospital, Washington, DC 20007, USA; Division of Hematology and Oncology, Department of Medicine, MedStar Washington Hospital Center, Washington, DC 20010, USA; Division of Endocrinology, Department of Medicine, MedStar Washington Hospital Center, Washington, DC 20010, USA

**Keywords:** 22.q11.2 deletion syndrome, DiGeorge syndrome, TBX-1, hypothyroidism, hypoparathyroidism

## Abstract

22q11.2 deletion syndrome (22.q11.2 DS) is a genetic syndrome resulting from a microdeletion on chromosome 22. It has a diverse array of manifestations, and most cases are diagnosed early in childhood. We present the case of a 38-year-old female born in a developing country who presented to our clinic to establish care for a history of primary hypothyroidism. She was clinically and biochemically euthyroid on thyroid supplementation. She was also noted to have hypocalcemia in the setting of low PTH, for which the patient was previously prescribed calcitriol. Given a history of cleft palate, abnormal facial features, mild recurrent sinopulmonary infections, and her endocrine history (including short stature with height in the 6th percentile), genetic testing was obtained. She was diagnosed with a heterozygous whole gene deletion of the *TBX1* gene. Additional genetic evaluation demonstrated a 2.6-Mb microdeleted segment of the 22a11.2 region encompassing 62 genes. The patient was referred to cardiology for evaluation of cardiac involvement given a history of tachyarrhythmia. This case highlights challenges in diagnosis and the implications of a delayed diagnosis of 22.q11.2 DS.

## Introduction

22q11.2 DS, also known as DiGeorge syndrome and velocardiofacial syndrome, is a genetic syndrome with a diverse phenotypic presentation. It results from a microdeletion on chromosome 22, referred to as 22q11.2 (1, 2). This region encodes over 90 genes, most notably T-box transcription factor 1 (*TBX-1*), which is responsible for the development of the pharyngeal pouches. Mouse and zebrafish *TBX1* knockout models demonstrate severe pharyngeal, cardiac, thymic, and parathyroid abnormalities, in addition to behavioral issues (3). Most microdeletions arise de novo, and parents of patients with 22q11.2 DS usually have no identifiable genetic aberrations (2, 4). The prevalence is roughly 1 in 4000 to 6000, though it is likely underreported due to lack of clinician recognition and lack of access to genetic testing (1, 5, 6).

Characteristic clinical features include thymic hypoplasia, cardiac, maxillofacial, endocrine, ocular, renal, gastrointestional, and skeletal abnormalities, autoimmune diseases, and neurodevelopmental and psychiatric diseases. As a result of thymic hypoplasia, patients may have a T cell immunodeficiency manifested by recurrent sinopulmonary infections. Fewer than 1% of patients have a complete absence of the thymus, which results in severe combined immunodeficiency and death within 12 months of age (1). In addition, a wide range of congenital heart defects (CHD) are present in 64% of patients and are the major cause of mortality (∼87% of all deaths) in children (2).

## Case Presentation

A 38-year-old female of South Asian descent with a history of hyperlipidemia, prediabetes, supraventricular tachycardia status post ablation, cleft palate status post repair, generalized anxiety disorder, hypothyroidism, and hypocalcemia presented to the endocrine clinic to establish care for her hypothyroidism. Hypothyroidism was diagnosed 2 years prior to her presentation, and the patient was treated with levothyroxine 125 mcg daily. Her history was notable for the diagnosis of hypocalcemia approximately 2 years prior to her presentation. She was prescribed calcitriol 0.25 mcg daily and 25-hydroxy vitamin D3 50 000 IU weekly. The patient reported an occasional history of transient perioral numbness but no history of paresthesia, carpopedal spasms, or seizures.

## Diagnostic Assessment

The patient’s physical exam was significant for short stature (152.4 cm, 6th percentile), microcephaly (head circumference of 48.5 cm, <1st percentile), mid-facial hypoplasia, a tubular and bulbous nose, and nasal voice. She had no cardiac murmurs. Chvostek and Trousseau sign were negative.

An initial workup of her hypocalcemia (with the patient on supplementation) was consistent with primary hypoparathyroidism: an ionized calcium of 4.3 mg/dL (1.07 mmol/L) (normal reference range: 4.5-5.6 mg/dL; 1.13-1.40 mmol/L), serum calcium 8.5 mg/dL (2.13 mmol/L) (normal reference range: 8.7-10.3 mg/dL; 2.17-2.58 mmol/L), serum phosphorous 4.6 mg/dL (1.49 mmol/L) (normal reference range: 3.0-4.3 mg/dL; 0.97-1.39 mmol/L), and an inappropriately low PTH of 12 pg/mL (1.27 pmol/L) (normal reference range: 15-65 pg/mL; 1.59-7.09 pmol/L). Despite mild hypomagnesemia of 1.5 mg/dL (0.62 mmol/L) (normal reference range: 1.6-2.3 mg/dL; 0.66-0.95 mmol/L), her PTH remained inappropriately low following magnesium repletion. Her serum creatinine was within normal limits. Serum 25-hydroxyvitamin D was 9.4 ng/mL (23.46 nmol/L) (normal reference range: 30.0-100.0 ng/mL; 74.88-249.6 nmol/L).

At our initial visit with the patient, who was already on thyroid supplementation, she was clinically euthyroid. Thyroid function tests showed a TSH of 0.577 mcIU/mL and a free T4 of 2.00 ng/dL (25.74 pmol/L) (normal reference range: 0.82-1.77 ng/dL; 10.55-22.78 pmol/L).

**Figure 1.**
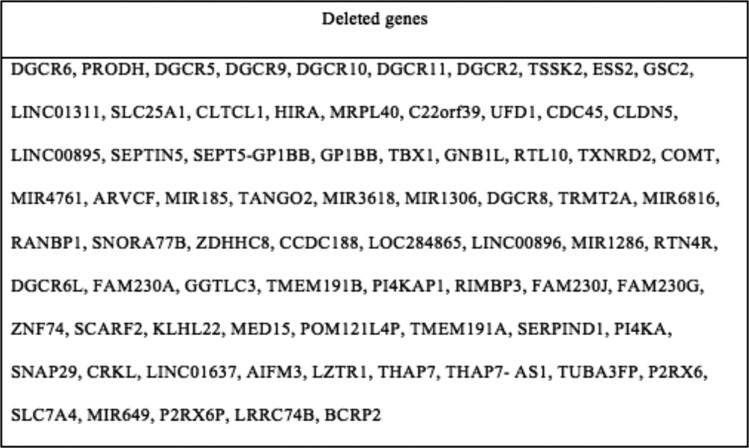
Genetic evaluation demonstrated a 2.6-Mb microdeleted segment of 22a11.2 region encompassing 62 genes, including the *TBX1* gene. Deletion of the *TBX1* gene is thought to be responsible for clinical manifestations of 22q11.2 deletion syndrome.

Given her history of primary hypoparathyroidism and hypothyroidism, further history was elicited given the association of these conditions with 22q11.2 DS. She reported a history of poor feeding as an infant, cleft palate status post repair, recurrent sinopulmonary infections, speech difficulties, and anxiety. As her presentation was concerning for 22q11.2 DS, she was referred to medical genetics. Chromosomal microarray analysis revealed a heterozygous 2.6-Mb deletion in chromosome 22 from position 18895006 to 21462658 (Fig. 1). This includes deletion of the *TBX1* gene, consistent with a diagnosis of 22q11.2 DS.

## Treatment

Her hypocalcemia regimen was kept at the preexisting dosage, but her levothyroxine was reduced to 100 mcg daily given a low-normal TSH value. Five months following dose reduction, TSH remained within normal limits at 3.09 mcIU/mL.

## Outcome and Follow-up

**Figure 2.**
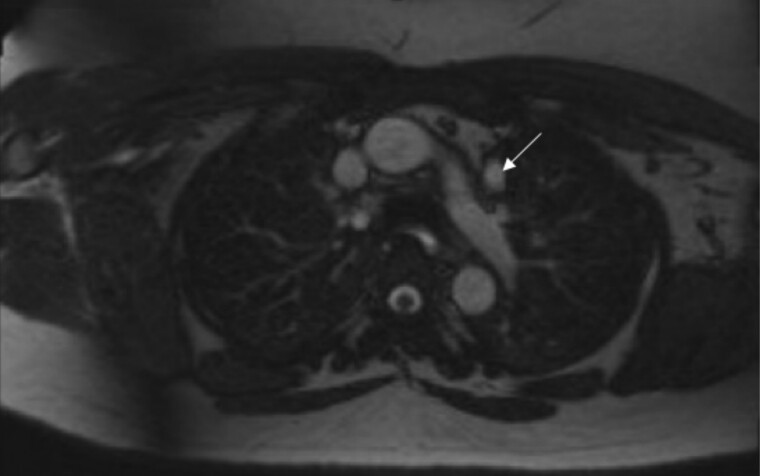
Axial magnetic resonance imaging of the chest shows a persistent left-sided superior vena cava (white arrow). The normal right-sided vena cava is also present.

Given the significant risk of cardiac anomalies and symptoms of palpitations and exertional dyspnea, the patient was referred to cardiology for further evaluation. Stress cardiovascular magnetic resonance imaging was obtained and revealed a persistent left superior vena cava with lack of bridging innominate vein, as well as a dilated aortic root of 38 mm at the sinus of Valsalva (Fig. 2).

**Figure 3.**
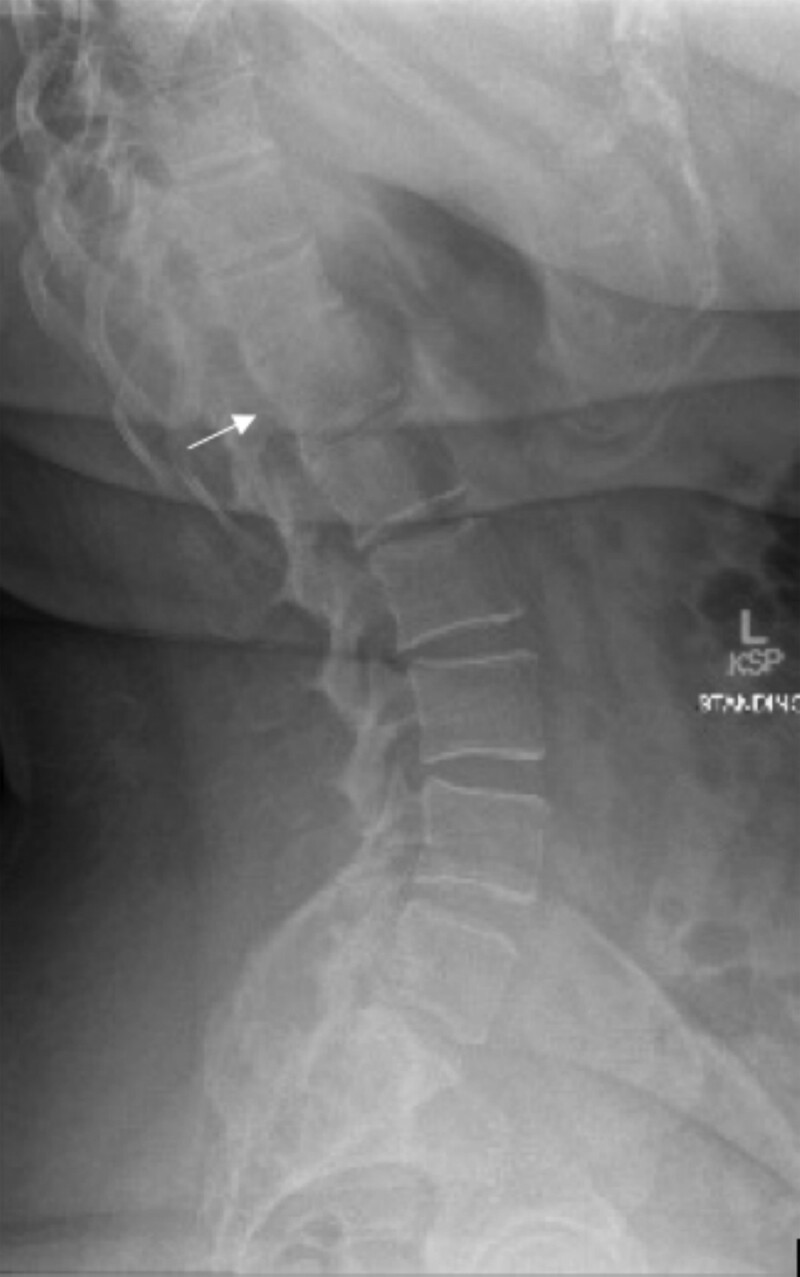
Lateral radiograph of the lumbar and thoracic spine revealed wedging of the T12 vertebrae (white arrow), consistent with a compression fracture of T12.

To complete her evaluation, she was given the following: testing of serum immunoglobulins (serum IgA, IgG, and IgM were within normal limits), referral to immunology, spine X-ray for kyphoscoliosis screening, and speech and language pathology for speech difficulties. Spinal x-ray incidentally noted a mild compression fracture of T12 (Fig. 3), though a dual-energy X-ray absorptiometry scan showed normal bone density. Evaluation for secondary causes of osteoporosis-—estrogen deficiency, iatrogenic (eg, glucocorticoids), or lifestyle contributions (eg, tobacco or alcohol)—was unrevealing with the exception of a presumed iron deficiency anemia. Laboratory results revealed a hemoglobin of 9.3 gm/dL (5.77 mmol/L) (normal reference range: 11.1-15.9 gm/dL; 6.89-9.87 mmol/L), microcytosis of 65 fL (65 µm^3^) (normal reference range: 79-97 fL; 79-97 µm^3^), and an elevated red blood cell distribution width of 17.3% (normal reference range 11.7-15.4%). In light of normal bone density, pharmacologic treatment for osteoporosis was not given. She is awaiting a workup of iron deficiency anemia.

## Discussion

While most patients are diagnosed during the neonatal period and early childhood, some may not be recognized until later into adulthood (1, 5, 7). Even in resource-rich countries, the condition is underdiagnosed, but this holds particularly true in developing nations and in non-Caucasian populations (6). For example, in a cohort of 156 Chinese adults with conotruncal heart defects, Liu et al found that 11.5% had previously undiagnosed 22.q11.2 DS. Furthermore, they found that while the clinical geneticist identified dysmorphic features in all patients, the cardiologist was only able to identify two-thirds of the patients as having dysmorphic facial features (5). Although these clinicians were cued specifically to search for features of 22.q11.2 DS, a significant number of patients were missed. This highlights the challenge in recognizing often subtle clinical and morphological features associated with this syndrome, especially for clinicians who may not have specialized training in recognizing these (5).

Another study by Kruszka et al found significantly variable phenotypic presentations among patients of different ethnicities (6), and most studies to date have focused on patients of European descent. This further complicates diagnosis in diverse populations. Interestingly, the authors proposed using facial analysis software as a diagnostic tool, noting that given its high sensitivity/specificity and the widespread use of mobile devices throughout the world, it could be a valid alternative to genetic testing in resource-limited settings (6).

Unfortunately, our patient remained undiagnosed until her late 30s, and her case highlights many of these disparities and challenges in diagnosis. She was born in a resource-poor setting and is of non-Caucasian descent. She had numerous syndromic manifestations of 22.q11.2 DS, which could have prompted an earlier diagnosis and timely evaluation by appropriate subspecialists. It is imperative for clinicians to maintain a high index of suspicion when characteristic manifestations are present but to also be aware of wide phenotypic variation, particularly in patients of diverse ethnic backgrounds.

The medical implications of timely diagnosis are important. For example, immunologic manifestations might require serial testing of vaccine titers and avoidance of live vaccines; irradiated blood products are often needed; and select patients need intravenous immunoglobulin and antibiotic prophylaxis (2). Furthermore, cardiac consequences are serious, with CHD representing the major cause of mortality in children (2). Adults with 22.q11.2 DS die prematurely, with sudden death and heart failure among the most common causes, even in patients without CHD (7). Guidelines recommend comprehensive evaluation by numerous physician subspecialists and ancillary providers (2).

Without a timely diagnosis, patients might not receive proper genetic or preconception counseling. While 90% of microdeletions arise de novo and only 10% of patients receive it from a heterozygous parent, the implications for offspring are significant (2). Offspring of affected individuals have a 50% chance of inheriting the deletion, and penetrance is nearly complete (2). Genetic testing of asymptomatic parents and siblings is recommended to identify people who would benefit from immunologic and cardiac evaluations. In the event of pregnancy, patients should be offered preimplantation genetic testing to mitigate the risk of inheritance to offspring, and fetuses at high risk should receive screening echocardiograms. Once born, affected neonates have high rates of cardiac decompensation and failure to thrive (8). From the maternal standpoint, affected pregnant patients might need closer monitoring. Thus, the implications of a missed diagnosis are significant for affected patients, their living family members, and their future offspring.

Endocrinologists should be cognizant of endocrine manifestations of 22.q11.2 DS, which include: hypocalcemia secondary to parathyroid aplasia or hypoplasia (∼70% prevalence) (9, 10), hypothyroidism (5-7% prevalence) (11, 12), Graves disease (13), and GH deficiency (∼41% prevalence) (13). Hypocalcemia is one of the cardinal features of 22.q11.2 DS. Calcium repletion targets the low-normal range, as PTH deficiency causes hypercalciuria with a high risk of nephrolithiasis (14). Patients are typically prescribed 1,25-dihydroxy vitamin D, with or without calcium supplementation.

Our patient's report of occasional, transient perioral numbness follows the typical pattern of hypocalcemia in 22.q11.2 DS. Severe hypocalcemia marked by tetany and seizures commonly occurs in the neonatal period but resolves soon thereafter, with intermittent recurrences (due to low parathyroid reserve) during increased physiologic stress or metabolic demand (14). Furthermore, there is a possibility her vertebral compression fracture was a manifestation of hypoparathyroidism. While well-described orthopedic manifestations of 22.q11.2 DS include anomalies such as kyphoscoliosis, polydactyly or syndactyly, and patellar dislocation, there is little data on the prevalence of fractures (15). Independent of 22.q11.2 DS, nonsurgically induced hypoparathyroidism is associated with increased fracture risk (16). While her compression fracture raises concern for fragility fracture (and thus osteoporosis), her bone density was normal, and factors other than bone density (eg, microarchitecture, mineralization, collagen structure) contribute to fracture risk (17). For these reasons, she was not treated with a bisphosphonate.

Her persistent left superior vena cava appears to be a rare manifestation of 22.q11.2 DS, though it is of unclear significance in her specific case (2, 18). Although in many patients this abnormality is incidentally diagnosed and patients remain asymptomatic, with certain anatomic circumstances it can lead to compression of the atrioventricular node and Bundle of His, causing cardiac arrhythmias. Awareness of the abnormal anatomy is also important during invasive procedures such as central venous line insertion (19).

In conclusion, 22.q11.2 DS remains an underrecognized entity with challenges in diagnosis, particularly in resource-poor settings and in patients from diverse ethnic backgrounds. Delayed diagnosis has important implications for the patient, their family, and their offspring. Although the clinical presentation of 22.q11.2 DS is remarkably diverse, it is important for clinicians to maintain a high index of suspicion in patients with syndromic presentations.

## Learning Points

- 22.q11.2 DS can present in almost every organ system, and most patients are diagnosed in early childhood.
- Patients from resource-poor settings or non-Caucasian populations often go undiagnosed. In part, this is due to lack of resources but is also due to wide phenotypic variation, particularly in patients of diverse ethnic backgrounds.
- A missed diagnosis of 22.q11.2 DS impacts affected patients, their living family members, and their future offspring.

## Data Availability

Original data generated and analyzed during this study are included in this published article.
